# *Cassia alata, Coriandrum sativum, Curcuma longa* and *Azadirachta indica*: Food Ingredients as Complementary and Alternative Therapies for Atopic Dermatitis-A Comprehensive Review

**DOI:** 10.3390/molecules27175475

**Published:** 2022-08-26

**Authors:** Yik-Ling Chew, Mei-Ann Khor, Zhao Xu, Sue-Kei Lee, Jing-Wen Keng, Sze-Huey Sang, Gabriel Akyirem Akowuah, Khang Wen Goh, Kai Bin Liew, Long Chiau Ming

**Affiliations:** 1 Faculty of Pharmaceutical Sciences, UCSI University, Kuala Lumpur 56000, Malaysia; 2 Faculty of Data Science and Information Technology, INTI International University, Nilai 71809, Malaysia; 3 Faculty of Pharmacy, University of Cyberjaya, Cyberjaya 63000, Malaysia; 4 PAP Rashidah Sa’adatul Bolkiah Institute of Health Sciences, Universiti Brunei Darussalam, Gadong BE1410, Brunei

**Keywords:** eczema, integrative medicine, herbal research, traditional medicine, complementary and alternative medicine, disease management

## Abstract

Traditional medicine is critical in disease treatment and management. Herbs are gaining popularity for disease management and treatment. Therefore, they can be utilised as complementary and alternative treatment (CAT) ingredients. Atopic dermatitis (AD) is one of the common non-communicable diseases. It is characterised by chronic inflammatory skin disease with intense pruritus and eczematous lesions. AD is associated with oxidative stress, microbial infection, and upregulation of inflammatory cytokines. Both children and adults could be affected by this skin disorder. The prevalence of AD is increasing along with the country’s level of development. This review revisited the literature on four medicinal herbs widely used as complementary medicine to manage AD. These therapeutic herbs are commonly eaten as food and used as spices in Asian cuisine. The four food herbs reviewed are *Cassia alata*, *Coriandrum sativum*, *Curcuma longa* Linn, and *Azadirachta indica*. Their traditional uses and phytochemical content will be covered. Four relevant pharmacological and biological activities of the plants crucial in AD management have been reviewed and discussed, including anti-inflammatory, anti-microbial, antioxidant, and wound recovery.

## 1. Introduction

Atopic dermatitis (AD) is a chronic inflammatory skin disorder that affects a particular population worldwide. It is characterised by pruritic, erythematous, scaly papules, vesicles, and plaques that might continue as chronic lichenified lesions caused by water loss through the epidermis, intense itching, and cutaneous inflammation [1,2]. The severe itching will cause excessive scratching, and it may cause an open wound. The skin of an AD patient has an altered skin microbiome, mainly colonised by *Staphylococcus aureus*. Skin infection happens when the microorganism enters the body via an open wound. Furthermore, excessive free radicals would mediate lipid peroxidation, leading to membrane destruction.

This skin disorder affects 60–80% of patients with a family medical history of type 1 allergies, allergic rhinitis, and asthma [3,4]. The patients have an inherited tendency to produce immunoglobulin E (IgE) antibodies in response to allergens. A defective skin barrier can easily allow the allergens to enter the epidermal and cause a secondary immunologic reaction. Allergens include dust, pollen, house dust mite, air pollutants, and even food allergens [5]. These allergens are generally concurrent with allergy, asthma, and stress [6]. This skin disorder is a significant social and financial burden to the patients, their families, and the whole community [5,7].

## 2. Current First-Line Therapy

Topical corticosteroids are the first-line therapy for AD. Topical corticosteroids could exhibit various vital activities which are mandatory in managing AD. It is effective in controlling skin disorders, and they are also fast-acting. However, prolonged use and exposure to topical corticosteroids could induce various side effects, such as local irritation, atrophy changes, telangiectasia, striae, purpura, and stellate pseudoscars hyperpigmentation, hypopigmentation, and rosacea [8]. Luger et al. [9] reported that 95.6% of AD patients use topical corticosteroids. A total of 50% of these patients were on topical corticosteroids daily for more than a year. Many patients rely on topical corticosteroids to manage this skin disorder daily. However, patients’ continuous exposure to topical corticosteroids may lead to excessive exposure compared to the recommended dosage in clinical practice and treatment guidelines [9]. Some patients have experienced steroid withdrawal symptoms after stopping the medication after prolonged use.

## 3. Use of Complementary and Alternative Treatment (CAT) in AD

In recent years, AD patients have begun to seek CAT to manage AD. Patients are switching to the CAT when they realise that topical corticosteroids’ side effects are irreversible and long-term. It was reported that almost 50% of the patients used CAT to manage the disease [10] (Table 1) and favoured plant-based ingredients in CAT [11,12]. This review emphasised medicinal herbs popularly used as CAT for AD. These therapeutic herbs are commonly served as food or spices in Asian cuisine. They are *Cassia alata* L., *Coriandrum sativum* L., *Curcuma longa* L., and *Azadirachta indica* A. Juss. These four medicinal herbs are widely consumed as food and spice. Still, they have also been used traditionally among the local communities in Asia, mainly in Southeast Asian countries such as Malaysia, Thailand, Indonesia and Brunei. One of the applications among the local community is skin disease management. The traditional usage, phytochemicals, and the core pharmacological activities of these herbs in managing AD were reviewed. The phytochemicals and pharmacological activities covered are anti-inflammatory, anti-microbial, antioxidant, and wound recovery. This review has supported evidence from relevant studies on the selected herbs in the management of skin disorders.

## 4. *Cassia alata* L. (Caesalpinaceae)

**Figure 1:**
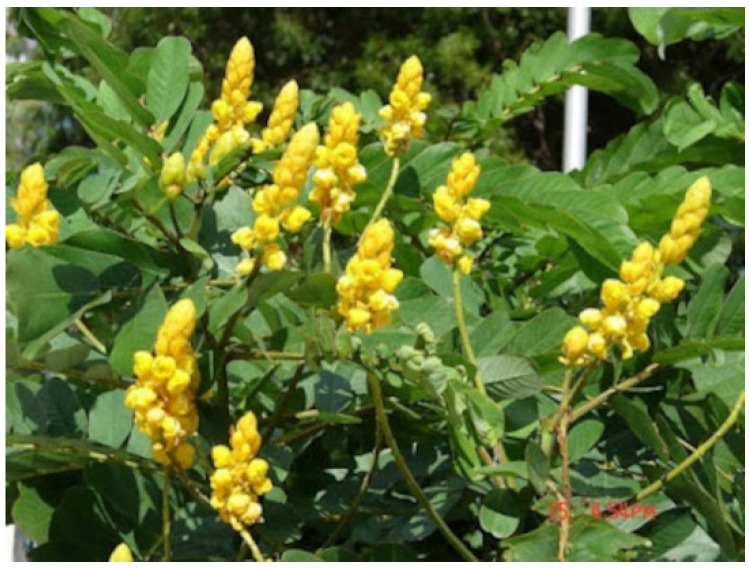
*Cassia alata* plant.

*Cassia alata* has been used traditionally as herbal therapy for various illnesses (Figure 1). The leaves are boiled and consumed for constipation and intestinal worm treatment. They are also prepared as a paste and applied topically to manage various skin disorders, such as AD, ringworm, and white-spot fungal infections [13,14,15]. The phytochemical ingredients in *C. alata* flower and leaves could exhibit promising antifungal and wound healing properties [13,16,17,18,19]. *C. alata* consists of various phytochemicals, including alkaloids, tannins, saponins, phenols, flavonoids, anthraquinones, and cardiac glycosides [20,21,22]. Astragalin, kaempferol, kaempferol 3-O-β-glucopyranoside, kaempferol 3-*O*-gentiobioside, rhein, aloe-emodin, emodin, β-sitosterol, 1,5-dihydroxy-2-methylanthraquinone, physcion, alatinone, n-octacosane, α-amyrin arachidate, tetracosanol, β-sitosterol, ursolic acid, and β-sitosterol-β-D-glucoside were among the phytochemicals reported in *C. alata* [23,24,25,26]. These phytochemicals played a significant role in pharmacological activities.

### 4.1. Anti-Inflammatory

*C. alata* had also exhibited excellent anti-inflammatory activity in various in vitro and in vivo models (Table 2). Anti-inflammatory activity was reported in the carrageenan-induced mouse paw oedema test when the mice were fed orally with *C. alata* extracts [18]. The inflammatory activity was inhibited at 5 mg/20 g. This finding is supported by Lewis et al. [27]. The authors discovered that *C. alata* leaves inhibited the production of tumour necrosis factor-alpha (TNF-*α*) by immature dendritic cells in a dose-dependent manner. TNF-*α* is an inflammatory cytokine produced during acute inflammation. *C. alata* leaves consist of TNF-*α* inhibitors which could suppress the physiologic response to inflammatory response [7]. The suppression of inflammatory response by the inhibitors is evident in the study by Riaz et al. [26]. Astragalin from *C. alata* could regulate the level of the anti-inflammatory related transcription factor, enzymes, and cytokines such as TNF-*α*, inducible nitric oxide synthase (iNOS), cyclooxygenase-2 (COX-2), prostaglandin E2 (PGE2), matrix metalloproteinase-1 (MMP-1), MMP-3, interleukin-1β (IL-1*β*), IL-4, IL-6, IL-8, IL-13, and interferon-gamma (IFN-γ). Anthraquinones and flavonoids in *C. alata* are good anti-inflammatory agents. Various studies have reported anti-inflammatory properties. Multiple studies had been performed on the anti-inflammatory of astragalin (Table 2). Besides, kaempferol, luteolin, apigenin, naringenin, rhein, chrysophanol, aloe-emodin, emodin and caffeic acid could also exhibited anti-inflammatory activity. The anti-inflammatory activity was targeted via various pathways: kaempferol inhibits the nuclear factor kappa-light-chain-enhancer of activated B cells (NF-kβ), mitogen-activated protein kinase (MAPK) and extracellular signal-regulated kinase (ERK) [28,29,30,31]; luteolin regulates the expression of NF-kβ, MAPK, signal transducer and activator of transcription 3 (STAT-3), iNOS, and COX-2 [32,33,34,35]; emodin suppresses IgE-mediated activation of mast cells, NF-kβ, and MAPK [36,37,38]; Aloe-emodin and apigenin inhibits iNOS, COX-2, protein kinase C, ERK, MAPK, NF-kβ, Janus kinase (JAK), c-Jun N-terminal kinases (JNK), and STAT-3 [35,39,40,41,42].

Many other related anti-inflammatory studies of *C. alata* using various in vitro and in vivo models were summarised in Table 2. Therefore, this plant is believed to inhibit the skin inflammatory response in AD effectively.

### 4.2. Antimicrobial

*C. alata* exhibits good antibacterial and antifungal activities toward a broad spectrum of pathogenic microorganisms [43]. These pathogenic microorganisms could cause severe skin infections and complications. Examples of bacteria that were susceptible to *C. alata* were *S. aureus* (including methicillin-resistant *S. aureus* (MRSA)), *Escherichia coli*, *Streptococcus pyogenes*, *Bacillus subtilis*, *Pseudomonas aeruginosa*, *Proteus mirabilis,* and *Vibrio cholerae* [20,44,45]. A recent study revealed that 9-octadecenoic acid methyl ester from *C. alata* leaves is a potent anti-microbial agent against MRSA [45]. The skin of AD patients is often over-colonised by bacteria, especially *S. aureus* [46]. Although *S.* *aureus* is a commensal inhabitant of the human skin, this bacterium could cause severe skin infection if it breaches the defective skin barrier and wound. It produces exotoxins with superantigenic properties. These exotoxins could lead to adverse effects on cell function and induce cell death.

The colonisation of *S. aureus* on atopic skin should be adequately controlled with anti-microbial agents to reduce skin inflammation. *C. alata* is effective in AD management because it inhibits the growth of *S. aureus* on the skin, reducing skin inflammation [20]. Topical treatment of skin inflammation with *C. alata*, like what has been used traditionally, showed that the skin conditions improved significantly. It has been proven by Breuer et al. [46], who discovered that 90% of the patients treated with anti-microbial agents, specifically targeting *S. aureus,* had a lower SCORing Atopic Dermatitis (SCORAD) score. A recent study revealed that the diethyl ether fraction of *C. alata* leaves was the most potent fraction against MRSA, eluted from column chromatography. The authors performed fractionation, and the antibacterial activity of the fractions was screened using the agar overlay method. The active spot was analysed using the gas chromatography-mass spectrometry (GC-MS) method. The spectrum has shown that the anti-microbial compound is 9-octadecenoic acid methyl ester [45].

The anti-microbial activity of the phytochemicals in *C. alata*, particularly towards *S. aureus,* was studied. Phenolic acids, flavonoids and anthraquinones in *C. alata* could exhibit promising anti-microbial activity. For instance, kaempferol and aloe emodin showed excellent anti-microbial activity toward multidrug-resistant *S. aureus* (MIC_50_ 13.0 ± 1.5 μg/mL and 12.0 ± 1.5 μg/mL, respectively). In comparison, kaempferol 3-*O-β*-glucopyranoside and kaempferol 3-*O*-gentiobioside exhibited less potent activity (MIC_50_ 83.0 ± 0.9 μg/mL and 560.0 ± 1.2 μg/mL, respectively) [1]. Hazni et al. reported that the free hydroxyl group at the C-3 position of kaempferol and its derivatives is essential in exhibiting anti-microbial activity. The size of the R1 group of the kaempferol derivatives would also affect the anti-microbial potency. The bigger the size of the group at the R1 position, the weaker the anti-microbial potency [1]. Gallic acid, caffeic acid, cannabinoid dronabinol, rhein and fatty acids in the leaves and seeds could also exhibit antibacterial activity against *S. aureus* [2,3,4,5].

Quercetin could also exhibit potent antibacterial activity. The activity is due to the multiple hydroxyl groups in the chemical structure [6,7,8,9], which could affect the peptides, proteins and ion channels in the bacterial membrane [8,10]. It could also disrupt the cell wall, weaken the bacteria cell membrane integrity and cause cell apoptosis [11]. Rhein could also exhibit potent antibacterial activity against *S. aureus* (MIC 4 µg/mL; MIC_90_ 8 µg/mL). It regulates the expression of transporter genes [12], changes the biological mechanism in the bacteria, such as inhibiting the aerobic and anaerobic respiration of bacteria and regulates the metabolism of the amino acid.

In addition, *C. alata* could also exhibit good anti-microbial action against pathogenic fungi related to skin disorders, including dermatophytes, such as *Aspergillus niger, A. flavus, A. candidus, Penicillium patulum, Candida albicans, Rhizopus stolonifera, Trichophyton mentagrophytes var interdigitale, T. mentagrophytes var. mentographytes, T. rubrum, Microsporum gypseum and M. canis* [47,48]. Therefore, the anti-microbial activity of *C. alata* has contributed significantly to skin disease management and is a prominent candidate in CAT.

### 4.3. Antioxidant

A high level of oxidative stress and reduction in antioxidants in the body system are important contributing factors in the pathogenesis of AD [49]. *C. alata* is rich in polyphenols which could exhibit strong scavenging activity against free radicals and oxidative agents, such as nitric oxide, hydrogen peroxide, superoxide anion, 1, 1-diphenyl-2-picrylhydrazyl (DPPH), and 2,2′-azino-bis(3-ethylbenzothiazoline-6-sulfonic acid)(ABTS) free radicals [47,50,51,52,53]. For instance, the extract inhibited 50% of the DPPH free radical at 2.25 ± 0.28 μg/mL. Its DPPH free radical scavenging activity was more potent than ascorbic acid (IC_50_ = 3.99 ±0.09 μg/mL) and Trolox (IC_50_ = 4.50 ±0.08 μg/mL) [13]. Besides free radical scavenging, it could exhibit good reducing power and lipid peroxidation inhibition effects [50,51]. Patients with AD usually will have higher serum malondialdehyde (MDA) levels. MDA is an oxidant provided by xanthine oxidase, which could cause lipid peroxidation. Redox imbalance due to excessive oxidative stress could also mediate the pathogenesis. A reduction in MDA was noticed in the Xanthine–Xanthine oxidase system, as reported by Sagnia et al. [52]. It was explained that the reduction in MDA was correlated with the reducing power. Phenolic and flavonoid content of the extract could exhibit good reducing power and hydrogen donation ability, and they play an important role in AD management. In a recent pilot clinical study, Sikora et al. developed a comprehensive, hydrating topical antioxidant product with *C. alata* and tested its efficacy on human [54]. Results showed that the product could protect the skin against induced oxidative stress due to exposure to harmful ultra-violet rays, due to the presence of water-soluble, enzymatic, and lipid-soluble antioxidants [54].

### 4.4. Wound Healing

Itching and redness are common signs of AD. Sometimes tiny bumps, blisters, or vesicles are noticed, and the skin is usually swollen. The itching on the skin would lead to excessive scratching, which may cause the skin to break and expose the open wounds. Local tissue damage will occur when the microorganism invades a wound and proliferates. The wound healing will also be disrupted.

Topical treatment with plant bioactive possesses wound recovery properties that would facilitate wound closure and recovery. *C. alata* was found could promote significant wound recovery [55]. An in vivo study by Kanedi et al. [56] showed the application of *C. alata* on an opened wound could reduce the epithelialisation period from 22 days to 16 days. The authors noticed that wounds inoculated with *T. rubrum* would require longer epithelialisation. However, anti-microbial agents in *C. alata* leaves could fasten wound recovery and epithelialisation. The wound recovery process was faster, and the epithelialisation period was shortened up to 27%. The size of the wound in the animal model was also reduced significantly. This result showed that the phytochemicals in the plant accelerated the wound healing process. One reason reported was that the inhibition of microbial infection promoted the epithelialisation process [57,58].

Alkaloids, terpenoids, flavonoids, anthraquinone and tannins could modulate wound closure. Oleic acid and linoleic acid could modulate the inflammatory responses in tissue repair, inhibit leukocytes and cationic serine protease activity, regulate the expression of metalloproteinase, and promote angiogenesis and collagen synthesis in wound recovery [13]. Wound healing is also closely associated with the anti-microbial activity of *C. alata*. 3,4- dihydroxycinnamic acid in *C. alata* has a broad spectrum of antibacterial activity, where it can fight Gram-positive and Gram-negative bacteria. These factors favour the healing process of wounds in patients [14].

## 5. *Coriandrum sativum* (Apiaceae)

**Figure 2:**
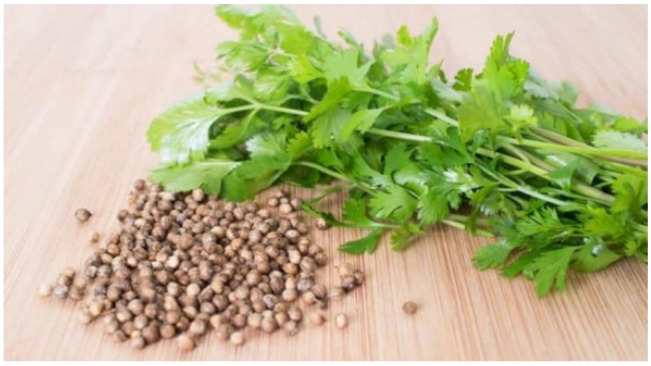
*Coriandrum sativum* plant and seeds.

*C. sativum*, commonly known as coriander, is a common herb (Figure 2). It is known as *Jintan, ketumar, ketumbar, penjilang, wansui* in Southeast Asia [59]. *C. sativum* is regularly consumed as spice and seasoning in local cuisines. However, it is also used in traditional medicine, where the plant is used to treat measles, indigestion, worm infections, rheumatism, pain in joints [60,61], and various skin disorders such as eczema, pimples, blackheads, dry skin, and skin ulcers [62,63,64,65]. It was reported that this plant could exhibit carminative, diuretic, tonic, stomachic, antibilious, anticatarrhal, antispasmodic, galactagogue, emmenagogue, and aphrodisiac properties [59].

*C. sativum* is rich in essential and fatty oils, 0.03–2.5% and 9.9–27.7%, respectively. *S-*(+)-linalool is the major essential oil in this plant. Other essential and fatty oil present include monoterpenes hydrocarbons *viz*. α-pinene, limonene, γ-terpinene, *p*-cymene, borneol, citronellol, camphor, geraniol and geraniol acetate, heterocyclic components like pyrazine, pyridine, thiazole, furan and tetrahydrofuran derivatives, isocoumarins, coriandrin, dihydrocoriandrin, coriandronsA-E, flavonoids, pthlides, neochidilide, digustilide phenolic acids, and sterols. The composition of essential oil in *C. sativum* was tabulated in Table 3.

### 5.1. Anti-Inflammatory

Significant anti-inflammatory activity had been reported in *C. sativum*, where it could alleviate contact dermatitis in the animal model. Sonika et al. [67] studied the anti-inflammatory activity of *C. sativum* ethanolic extract using carrageenan-induced rat paw oedema. A total of 40.81% oedema inhibition was reported in mice fed orally with a 200 mg/kg extract. Similar findings were also reported by Reuter et al. [62]. The authors noted that the anti-inflammatory activity of *C. sativum* was exhibited by a major fatty acid, linalool (65–75% in oil composition). It could regulate inflammatory mediators such as IgE, TNF-*α*, IFN-γ, IL-1, IL-4, and IL-13 [68,69]. In addition, it was found that *C. sativum* could reduce IL-6 [69]. A recent review by Malek Mahdavi and Javadivala [69] critically reviewed the anti-inflammatory activity reported in *C. sativum*, primarily focused on IL-1*β*, IL-6, and TNF-*α*. Various research using the in vitro and in vivo models agreed that *C. sativum* could exhibit promising anti-inflammatory activity. The expression of IL-1*β*, IL-6, and TNF-*α* was suppressed or lowered upon treatment with *C. sativum* extract, oil, and fractions. The suppression of inflammatory mediators was shown in the in vitro models such as lipopolysaccharide (LPS)-stimulated neutrophils [70], LPS-induced BV-2 microglia cells [71], LPS-stimulated RAW264.7 macrophage cells [72,73], and human bronchial epithelial cells [74]. The anti-inflammatory activities of *C. sativum* had also been investigated using various in vivo models. For instance, Freund’s adjuvant (CFA)-induced arthritic rats [75,76,77], ischemia/reperfusion injury (IRI) rats [78], and streptozotocin (STZ)-induced diabetic mice and rats [79,80]. However, none of the in vitro and in vivo models studied could be correlated with AD. The anti-inflammatory using AD models should be investigated.

The regulation of inflammatory mediators is critical in managing skin inflammation. Besides, it could also exhibit promising anti-microbial activity toward the *S. aureus* colonised on the skin [53].

### 5.2. Anti-Microbial

Numerous studies have reported on the anti-microbial activity of *C. sativum*. Linalool and coriander oil exhibited strong anti-microbial activity towards yeast, Gram-positive, and Gram-negative bacteria. Its activity was more potent than phenol. The inhibition activity against Gram-positive bacteria was two times stronger. In addition, the activity was 1.5 times more potent against yeast and Gram-negative bacteria [81]. Linalool in coriander could exhibit a broad spectrum of anti-microbial activity towards numerous microbial, including *S.* *aureus*, *Salmonella typhimurium*, *Listeria monocytogenes*, *Serratia grimesii*, *Enterobacter agglomerans*, *Yersinia enterocolitica*, *B. cereus*, *B. megaterium*, *Klebsiella pneumoniae*, *P. aeruginosa*, *E. coli*, *E. cloaca*, and *Enterococcus faecalis* [82,83]. Besides being an anti-microbial agent for skin disorders, coriander oil is also a penetration enhancer. It could alter the conformational domain of the skin and enhance transdermal drug delivery. Therefore, it could be added to the topical formulation for skin disease management and inhibit the skin microbial that cause skin infection.

### 5.3. Antioxidant

Numerous studies reported that the polyphenols, flavonoids, and terpenoids in *C. sativum* exhibited strong free radical scavenging and lipid peroxidation inhibition activities [61,84,85,86]. Twenty-one antioxidant compounds were reported in the recent study by Msaada et al. [87]. The compounds identified were mainly phenolic acids and flavonoids. The phenolic acids present were gallic, chlorogenic, caffeic, vanillic, *p*-coumaric, ferulic, rosmarinic, o-coumaric, trans-hydroxycinnamic, salicylic, and trans-cinnamic acids; while the flavonoids reported were quercetin-3-rhamnoside, rutin trihydrate, luteolin, quercetin dihydrate, resorcinol, kaempferol, naringin, apigenin, flavone, and coumarin. Chlorogenic acid and gallic acid are the major compounds present in *C. sativum*. It is rich in essential oils. Chlorogenic acid, gallic acid and essential oils could exhibit good to moderate antioxidant activity [88]. Hajlaoui et al. [89] recently studied the anti-microbial activity of the essential oil extracted from *C. sativum*. The essential oil could exhibit moderate DPPH scavenging (IC_50_ = 38.83 ± 0.70 µg/mL), superoxide anion scavenging (IC_50_ = 37.00 ± 1.73 µg/mL), reducing power (EC_50_ = 24.00 ± 1.53 µg/mL) and β-carotene bleaching (IC_50_ = 25.70 ± 1.02 µg/mL) activities. Other studies also support the free radical scavenging activity of coriander oil. Shahwar et al. [90] and Kačániová et al. [86] reported that the coriander oil could inhibit more than 50% of the free radicals in the assay. The essential oil could also exhibit chelating activity (EC_50_ = 70.00 ± 0.81 µg/mL) [89].

### 5.4. Wound Healing

Excessive scratching may cause the patches of skin to become red and scaly. Sometimes, an open wound will form. *C. sativum* has traditionally been used for wound healing due to its antiseptic and anti-microbial properties. It had been reported that *C. sativum* showed promising wound healing effects in second-degree burn wounds in the rat model. Zadeh et al. [91] studied the effect of wound healing cream formulated with coriander extract. The effects were compared with silver sulfadiazine cream and Vaseline gauze dressing applied to the burn wound in the rat. The results showed that the wound in the rats improved significantly. The authors explained that this was due to fatty acids, which could exhibit anti-inflammatory and anti-microbial activities. The fatty acids which exhibit prominent biological activities, such as oleic acid, linoleic acid, and palmitic acid, could effectively promote wound healing [92]. They accelerated tissue repair mechanisms by modulating the cellular response, increasing the migration of endothelial cells, and inducing angiogenesis at the wound site [93]. Consequently, the wound healed faster.

## 6. *Curcuma longa* Linn (Zingiberaceae)

**Figure 3:**
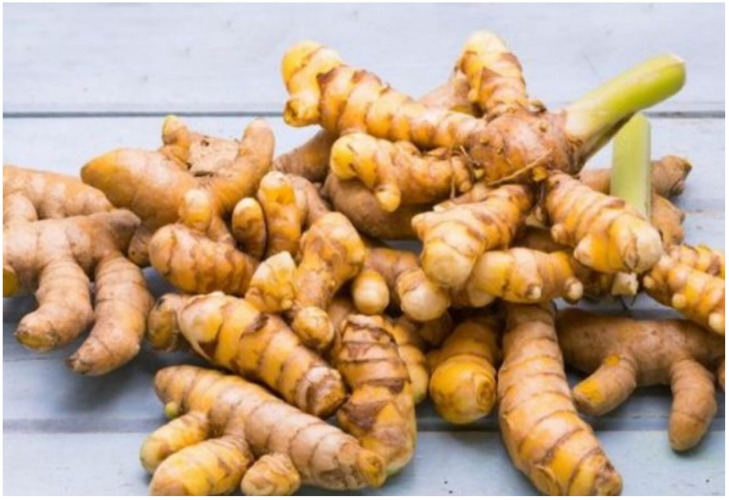
*Curcuma longa* rhizomes.

*C. longa*, often known as turmeric (Figure 3), is commonly used as a spice in Asian cuisine [94]. The plant has various common name in different locations, i.e., *Curcuma* (Sp. It. Fr.), *acafrao da India* (Portugal), *geelwortel* (Dutch), *kurkum* (Arab), *Manjano* (East Africa [KiSwahili]), *manjal* (Tamil), *kunyit* (Indonesia), *temu kunyit* (Asian), and *huang jiang* (Chinese). The ethnomedicinal use of *C. longa* has been known for years. It has been well documented in various literature due to its antioxidant, anti-inflammatory, antimutagenic, anti-microbial, and anticancer properties [95,96].

*C. longa* is commonly used in Ayurvedic and Unani systems of medicine [97]. In Ayurveda, fresh turmeric is processed into a paste to treat common eye infections, applied on a wound as a dressing, and treat bites, burns, acne, and various skin diseases. Turmeric milk with honey is consumed to reduce inflammation, and health maintenance, improve memory, prevent heart diseases, reduce the risk of cancer, regulate blood sugar levels, and many more. The poultice of turmeric is applied to the perineum to aid in healing any lacerations in the birth canal [98]. *C. longa* rhizome has traditionally been used in skin disorders management [83], dental diseases, dyspepsia, acidity, indigestion, flatulence, ulcers, antioxidants, antifertility, and alleviating the hallucinatory effects of hashish, and other psychotropic drugs [99].

*C. longa* is rich in polyphenolic curcuminoids, namely curcumin (80%), demethoxycurcumin (12%), bisdemethoxycurcumin (8%), and essential oil (5.8%) [100,101]. The essential oil which is present in rhizomes includes α-phellandrene (1%), sabinene (0.6%), cineol (1%), borneol (0.5%), zingiberene (25%), and sesquiterpenes (53%) [102]. These phytochemicals could exhibit important pharmacological activities, including anti-inflammatory, anti-microbial, antioxidant, and promote wound healing.

### 6.1. Anti-Inflammatory Activity

As mentioned, *C. longa* possesses significant anti-inflammatory activity. Curcumin in *C. longa* could exhibit strong anti-inflammatory action, and its potency is comparable to cortisone. It can inhibit the biosynthesis of inflammatory prostaglandins from arachidonic acid and neutrophil function during inflammatory states [103]. Prostaglandins and leukotrienes are the inflammatory mediators produced in the arachidonic acid oxygenation pathway. They contribute to common inflammatory responses, such as erythema, oedema, and pain. The reduction of prostaglandin and inhibition of leukotriene biosynthesis through the lipoxygenase pathway is crucial in controlling inflammation [104]. The inhibition activity of curcumin in the topical cream is proven effective in managing skin inflammation and allergies.

In addition, curcumin inhibits the synthesis of inflammatory-related cytokines, such as lipoxygenase (LOX), COX, phospholipases, leukotriene, prostaglandins, thromboxane, nitric oxide elastase, hyaluronidase, collagenase, monocyte chemoattractant protein-1, interferon-inducible protein, TNF-*α*, IL-12, IL-1α and IL-6 [105,106]. Studies have reported that curcuminoid could reduce the nitric oxide synthase in the animal model and inhibit inflammation. The amount of nitrite released into the culture medium in 24 h (IC_50_ = 6 µM) decreased significantly upon the treatment with curcumin. Nuclear Factor kappa-light-chain-enhancer of activated B cells (NF-*k*B) is highly activated at the inflammation site in AD. Curcumin can inhibit NF-*k*B to regulate the inflammation responses [106]. It could also induce the transcription of proinflammatory cytokines and regulate the activation and differentiation of inflammatory T-cells [105]. These anti-inflammatory activities would reduce the swelling skin reaction.

### 6.2. Anti-Microbial Activity

AD is often associated with dermatophyte infection. Dermatophytes hydrolyse keratin from skin tissue and cause superficial infections. A recent study by Choi et al. [107] reported that 1.42% of AD patients showed positive towards dermatophytes on their skin, whereas none was detected in healthy individuals. *C. longa* and curcuminoids have shown promising anti-microbial activity against various dermatophytes and pathogens, including *Fusarium miniformes* MAY 3629, *B. subtilis* ATCC 6633, and *F. oxysporum* ATCC 48122, *Plasmodium falciparum* and *Leishmania major, T. longifusus* and *Microsporum canis* and *S. aureus* [108,109,110]. Tumeric oil extracted from the plant is commonly used as a topical oil for treatment as it could exhibit good anti-microbial activity. The anti-microbial activity of turmeric oil was exhibited by aromatic tumerone. The activity was evaluated using guinea pigs. It was found that turmeric oil could inhibit all 15 dermatophytes isolates in the in vivo model. The skin microbial was inhibited at 1:40 to 1:320 dilutions of the turmeric oil [111]. In addition, the oil could also inhibit the other pathogenic fungi at 1:40 to 1:80 dilutions. The study showed that the skin lesion disappeared after seven days post-turmeric application. Therefore, it is deduced that *C. longa* is effective in managing AD.

Curcumin in *C. longa* could also exhibit synergistic effects with other phytochemicals. Curcumin could exhibit synergistic effects with quercetin. The co-delivery of curcumin and quercetin could exhibit anti-microbial activity against MRSA at lower concentration [112]. This is in agreement with Chittasupho et al., where the combination of curcumin with quercetin would exhibit antibacterial activity against *S. aureus* and *P. aeruginosa*, but not in individual compounds [113].

### 6.3. Antioxidants

The hepatoprotective effect of the ethanolic extract of *C. longa* on thioacetamide-induced liver cirrhosis had been correlated with its antioxidant effect and free radical scavenging activities. The hepatoprotective activity is proven to be contributed by curcumin in *C. longa*. Curcumin could also increase glutathione levels and promote the conversion of fat-soluble toxins into water-soluble toxins. These toxins would then be eliminated via liver detoxification [114]. In addition, tetrahydrocurcumin in the aqueous extract functions as an antioxidant in *C. longa* [115,116]. Sugiyama et al. [117] reported that tetrahydrocurcumin is a potent antioxidant. It could exhibit strong antioxidant activity and is more potent than curcumin. It scavenges and neutralises free radicals generated during skin inflammation and oxidative stress. Oxidative stress could cause the upregulation of proinflammatory cytokines. The cytokines would lead to the release of excessive free radicals and lead to the pathogenesis of AD. Therefore, antioxidants in *C. longa* would downregulate the proinflammatory cytokines, reduce the oxidative stress, scavenge the free radicals released and thus facilitate the skin barrier recovery.

### 6.4. Wound Healing

Wound healing is essential in managing AD as the open wound allows bacteria to enter the skin, leading to bacterial infections. The effect of *C. longa* in wound healing activity has been proven using the excision wound model. Curcumin in *C. longa* has been proven to exhibit wound healing potential. The wound contraction was more significant in the treated group, and the activity was positively correlated with *C. longa* extract [118]. Kulac et al. [119] studied the wound healing effects of topical curcumin therapy on a burnt rat model. The results revealed that curcumin in *C. longa* is effective in upregulating the expression of proliferating cell nuclear antigen in the skin tissues of rats in the burnt group. Maghima et al. [120] tested the wound recovery of the metallic silver nanoparticles (AgNPs) with *C. longa* leaf (CL-AgNPs). The study revealed that CL-AgNPs exhibited wound healing ability on fibroblast (L929) cells. A significant increase in cell migration towards the wounded region of the damaged fibroblast cells upon the treatment with CL-AgNPs [120]. Another study also supported that *C. longa*-treated burn wounds took a shorter time to heal, showing significantly higher collagen deposition, vascularisation, angiogenesis, and fibrosis during the healing process [121]. Curcumin is a proangiogenic mediator which could stimulate the transforming growth factor-beta [106]. It could result in the production of extracellular matrix and angiogenesis. Angiogenesis is the process of increasing the formation of blood vessel density. A higher rate of endothelial cell proliferation showed better progress in wound repair and closure [106]. Therefore, it is believed that *C. longa* could manage the skin condition of AD patients by promoting the wound healing process. This repairs and restores the damaged skin barrier.

## 7. *Azadirachta indica* A. Juss. (Neem) (Meliaceae)

**Figure 4:**
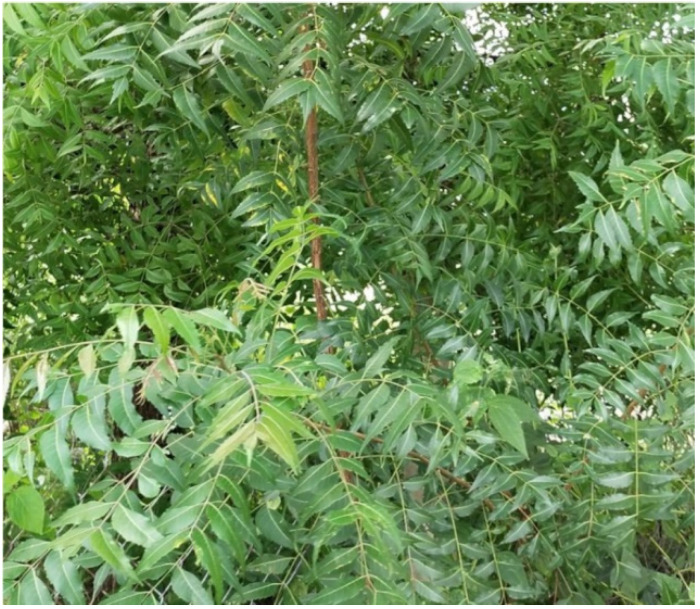
*Azadirachta indica* plant.

*A. indica*, commonly known as neem (Figure 4), grows in a broad region in tropical and semitropical countries such as India, Pakistan, Nepal, and Bangladesh. Neem extracts are commonly applied as complementary medicine in Ayurveda, Unani, and homoeopathy. These extracts have been reported to treat numerous skin disorders, including eczema, psoriasis, lice, ulcerated skin, and syphilitic sores [62,122].

Neem is rich in phytochemicals, and they possess valuable therapeutic functions. Various important bioactive compounds are found in multiple parts of the plant, and these phytochemicals are from triterpenes, flavonoids, limonoids, saponins, phenolic acids, and tannins. Examples of phytochemicals reported include azadirachtin, nimbolinin*,* nimbin, nimbidin, nimbidol, sodium nimbinate, gedunin, salannin, quercetin, nimbin, nimbanene, 6-desacetylnimbinene, nimbandiol, nimbolide, ascorbic acid, n-hexacosanol and amino acid, 7-desacetyl-7-benzoylazadiradione, 7-desacetyl-7-benzoylgedunin, 17-hydroxyazadiradione, and nimbiol. The seeds contain valuable constituents, including gedunin and azadirachtin [123]. Neem oil is also full of beneficial fatty acids. The major fatty acid present in neem oil is oleic acid (25–54%), followed by hexadecenoic acid (16–33%), stearic acid (9–24%), linoleic acid (6–16%), and a trace amount of alpha-linolenic acid and 9-hexadecanoic [124]. These fatty acids could exhibit various pharmacological activities that are extremely important in managing AD, such as antioxidant, anti-microbial, and anti-inflammatory activities.

### 7.1. Anti-Inflammatory

The anti-inflammatory activity could be achieved by regulating pro-inflammatory enzyme activities, including COX and LOX [123]. Chattopadhyay et al. [125] investigated the anti-inflammatory activity of neem tree extract in rats. It was found that significant anti-inflammatory activity was shown in the cotton pellet granuloma assay in the rat model at 200 mg/kg p.o. of ethanol leaves extract. Reductions in various biochemical parameters were noticed, including deoxyribonucleic acid, ribonucleic acid, lipid peroxide, acid phosphatase, and alkaline phosphatase in the rat. The extract has also been found to suppress exudate in the cotton pellet-induced model of inflammation in rats, and this showed the inflammation had been inhibited.

Another study by Mosaddek et al. [126] also supported that neem has good anti-inflammatory activity. 400 mg/kg body weight of aqueous extract of neem was given to the rats intraperitoneally once daily for seven days, one hour before the induction of paw oedema. The paw oedema was induced in the right hind paw of rats using 2% formalin in 0.9% NaCl subcutaneously. The circumferential length was measured before and after the formalin injection. The mean increase in the circumferential length of the hind paw oedema was calculated to determine the inflammatory exudative lesion and the percentage of inhibition. Authors reported that the increase in circumferential lengths was inhibited by neem extract due to its anti-inflammatory activity. Inhibition was also noticed in samples treated with the positive control, dexamethasone. After seven days of treatment, the circumferential length decreased from 15.5 ± 0.4 mm to 4.8 ± 0.3 mm for neem extract (400 mg/kg). Although the anti-inflammatory effect of neem extract was not as promising as dexamethasone (reduction from 15.5 ± 0.4 mm to 3.2 ± 0.6 mm for 0.75 mg dexamethasone), it was significantly better than the control group (reduction from 16.4 ± 0.4 mm to 9.2 ± 0.4 mm). Nimbidin and lupeol in *A. indica* were suggested to be one of the contributors to the anti-inflammatory activity [127]. Studies have reported that nimbidin could suppress the mechanism of action of macrophages and neutrophils associated with the inflammatory response [128].

Another investigation was conducted to study the anti-inflammatory effect of neem seed oil (NSO) on albino rats using carrageenan-induced hind paw oedema. The results revealed that NSO showed more significant inhibition of paw oedema with the increasing dose from 0.25 mL/kg body weight to 2 mL/kg body weight. At the highest amount (2 mL/kg body weight), NSO showed maximum (53.14%) inhibition of oedema at the 4th hour of carrageenan injection [129]. The finding is also supported by Ilango et al. [130]. The authors had isolated azadiradione, a triterpenoid, from the neem fruit. The anti-inflammatory activity of the fruit extract and azadiradione were determined using the carrageenan-induced paw oedema model. Significant anti-inflammatory activity was noticed at 100/mg/kg dose of the extract and azadiradione.

### 7.2. Anti-Microbial

Numerous studies reported that neem exhibited significant anti-microbial activity towards various bacterial and fungal strains. Anti-microbial activity of *A. indica* had been performed on *Salmonella typhi, E. coli V. cholerae,* and *B. subtilis* using agar well diffusion. The zone of inhibition measured was 10–17 mm [131]. Neem oil from the leaves showed a wide range of antibacterial activity against various bacterial and fungal strains, including *Staphylococcus* sp., *E. coli*, *B. cereus*, *Proteus vulgaris*, *S. typhimurium*, *K. pneumoniae*, *Shigella dysenterae*, *F. oxysporum*, *A. flavus*, *A. fumigates*, *A. niger*, *C. albicans*, *Cladosporium* sp., *M. canis*, *M. gypseum*, *T. rubrum*, *T. mentagrophytes*, and *Penicillum notatum* [124]. Patankar et al. [132] developed a hand sanitiser using neem leaf extract. The anti-microbial assays showed that it is effective against *S. aureus* and the antibiotic-resistant strains, which are the common bacteria causing AD. The discovery of the anti-microbial effect of neem is essential for developing new pharmaceutical products to meet the anti-microbial therapeutic needs [124]. Nimbidin, nimbin, nimbolide, Azadirachtin, gallic acid, epicatechin, catechin, and margolone had been reported to be the antibacterial compounds present [133]. The findings on the anti-microbial activity of neem are closely relevant to AD management, as this skin disorder is often associated with bacterial and fungal infection on the skin, especially when there is an open wound due to continuous scratching.

### 7.3. Antioxidant

Numerous antioxidants had been reported in *A. indica*, including gallic acid, epicatechin, catechin, and rutin [127,133]. Chronic skin inflammation is associated with the overproduction of free radicals, including reactive oxygen species, such as superoxide and hydrogen peroxide. The increase in free radicals in the cell could cause the cell to reduce in a protective capacity. It showed pathological states where the decrease in antioxidant activity could cause the weakening of the antioxidant mechanism, which could intensify the inflammatory condition of the skin [134,135]. Oxidative injury is also the fundamental mechanism that causes inflammation. The free radicals can also cause defective basement membrane associated with AD.

The antioxidant effect of neem has been studied. The total phenolic content (85.9 ± 4.0 mg gallic acid equivalent/g of plant extract), total flavonoid content (104.9 ± 5.5 mg rutin equivalent/g of plant extract), total anthocyanin content (65.3 ± 13.9 mg rutin equivalent/g of plant extract) and lipid peroxidation inhibition (50–70% inhibition at 0.2 mg/mL concentration) of the extract were evaluated and reported. [136]. The bark and leaf extracts could also exhibit promising radical scavenging activities [137]. The DPPH radical scavenging was found to increase with the increasing concentration of the extracts [134]. 70% of DPPH radical was inhibited by the leaf extract (200 µg/mL). In addition, neem also showed promising ferric ion reducing antioxidant activity (315.25 ± 23.81 mg/g), which measures the plant phytochemicals’ ability to reduce ferric ions Fe^3+^ to ferrous ions Fe^2+^.

### 7.4. Wound Healing

The wound healing effect of neem extract was studied using the scratch assay and excision and incision wound model in Sprague Dawley rats [138,139]. The extract demonstrated significant wound healing ability in both in vitro and in vivo models [138,139]. In the in-vitro scratch assay, the effectiveness of neem in promoting wound closure was evaluated. The thickness of the scratch was measured over 48 h, and the percentage of wound closure in keratinocytes and fibroblasts was also observed. The wound closure of keratinocytes and fibroblasts treated with neem was better than the control group. The wound closure was 85% at 24 h and almost 100% at 48% [139]. Promising wound healing activity was also noticed in the in vivo model. Up to 95% of the wound was healed on day 21, and the wound was completely healed on day 28. The wound healing ability was similar to the positive control, Himax ointment for the animal wound. The wound healing effect was significantly better than the control group, where the wound healing effect achieved only 85% on day 28 [138]. Another study by Osunwoke et al. [120] also supported that leaf extracts promote wound healing by increasing the inflammatory response and neovascularisation [140]. A significant percentage of wound contraction, fibroblast and blood vessel count were observed. No hypertrophic scars were noticed in the wound. The neovascularisation could increase wound tensile strength and promote healing [140]. The aqueous neem leaves extract also been studied on the wound healing effect in psoriasis skin disorder in patients. In the double-blind clinical trial, the patients with psoriasis were subjected to neem leaves extract treatment. The wound healing effect of neem was shown to be effective compared to the placebo group. It was reported that β-sitosterol in *A. indica* aid in wound healing. These findings would support the open wound in AD due to excessive scratching to heal effectively.

## 8. Conclusions

CAT is an essential practice in the country to improve the health and well-being of people. It has risen in the past decade, and many patients opt to visit CAT professionals. Herbs, herbal materials, preparations, and finished herbal products containing active ingredients, plant materials, extracts, or combinations are commonly used to manage skin disorders. Herbal medicine usage is popular among individuals and primary health care providers, such as traditional medicine practitioners. This niche area will become an essential component in integrative medicine that will improve health and quality of life with modern treatments.

## Figures and Tables

**Table 1:** Types of complementary and alternative medicine (CAM) used in children with AD at a tertiary care centre ^a^. Reproduced from Koo, Nagayah, Begum, Mahmood, and Shah [10].

Types of CAM	Percentages (%)
CAM user	46.8
**Traditional Malay Medicine**	
Malay herbs	13.9
Malay massage	0.6
Malay cupping	0.6
**Traditional Chinese Medicine**	
Chinese herbs	5.8
Islamic Medical Practice	
Ruqyah	16.2
Homoeopathy	9.2
Chiropractic	0.6
**Complementary Therapy**	
Spa therapy	0.6
Aromatherapy	1.7
Nutritional therapy ^b^	9.8
Others	35.3
Virgin coconut oil	11.0
Vitamin C	9.2
Olive oil	6.4
Prebiotic/Probiotic	2.3
Remdii™ ^c^	2.3
Oiling ^d^	1.7
Multivitamin	1.2
Omega oil	1.2
Blackseed oil	1.2
Redoxamin ^e^	1.2
Cetaphil^® f^	1.2
Honey	1.2
Others ^g^	11.6

^a^ Child may have used more than one form of CAM ^b^ Nutritional therapy includes organic diet, diet control, gluten-free, and avoidance of dairy food. ^c^ Remdii™—tocotrienol-enriched cream. ^d^ Oiling—any forms of oil not stated by the parents. ^e^ Redoxamin—a product that contains pineapple juice mixed with vitamin C and bromelain. ^f^ Cetaphils^®^—proven dermatological skincare. ^g^ Others include Scott’s^®^, 4Life^®^, Elken™, Amway™, Yakult^®^ (probiotic drink), Caliph™ (mix fruit extract drink), Al-Manna (Arabic gum), air zamzam (mineral water), ColoSkim by Zhulian™ (skim milk and colostrum mix), turmeric, cendawan kering (dry mushrooms), kurma kering (dry dates), hypoallergenic shampoo/shower, vaseline, aloe vera, CeraVe^®^ cream, Skin Ease^®^ cream, DIY Shea Butter, Nano Colloidal Silver (solution containing nanometre sized particles of suspended silver), herbal cream.

**Table 2:** Anti-inflammatory activities of astragalin in vitro and in vivo. Reproduced from Riaz et al. [26]. Table reproduced is under Creative Commons Attribution License.

Assays	Organism Tested	Dose/Concentration	Molecular Targets
LPS-induced mouse mastitis	Mouse mastitis	10, 25 and 50 mg/kg	TNF-*α* ^↓^, IL-1*β* ^↓^, IL-6 ^↓^, p65 ^┴^, and I*κ*B*α* ^┴^
LPS-induced endotozemia and lung injury in mice	Mice (lung)	25, 50, and 75 mg/kg	TNF-*α* ^┴^, IL-1*β* ^┴^, and IL-6 ^┴^
LPS-induced macrophages in mice	Mouse cells	1–100 μg/mL	IL-6 ^↓^, MIP-1*α* ^↓^, MCP-1 ^↓^, NF-*κ*B p65 ^┴^, I*κ*B*α* ^┴^, and NO ^┴^
LPS-induced RAW 264.7 cells	Mice (RAW 264.7 cells)	1, 10, and 100 μM	NO ^↓^ and TNF-*α* ^↓^
Inhibitory activity on the histamine release by KU812 cells	KU812 cells	10 to 30 μmol/L	IL-4 ^↓^, IL-13 ^↓^, and (IFN- γ) no effect
LPS-induced Inflammation in RAW 264.7 cells	Mice (RAW 264.7 cells)		NO ^┴^, IL-6 ^┴^, and PGE2 ^┴^
*Porphyromonasgingivalis*-induced human gingival epithelial (HGE) cells	Human gingival epithelial cells		COX-2 ^┴^, IL-6 ^┴^, IL-8 ^┴^, MMP-1 ^┴^, MMP-3 ^┴^, PGE-2 ^┴^, and IL-4 ^┴^
Anti-inflammatory effects on *Leptospira interrogans*-induced inflammatory response	Uterine and endometrial epithelial cells of mice	100 μg/mL	p38 ^┴^, p-p38 MAPK ^↓^, ERK ^┴^, JNK ^┴^, and p-p65 ^↓^
Protective effects against ovalbumin- (OVA-) induced allergic inflammation	Mouse model of allergic asthma	0.5 mg/kg and 1 mg/kg	SOCS-3 ^┴^, SOCS-5 ^┴^, and IFN- γ ^↑^
Alleviation in hepatic fibrosis function	Diabetic rats and nondiabetic		PAR2 ^┴^, IL-1*β* ^↓^, IL-6 ^↓^, TNF-*α* ^↓^, and TGF-*β*1 ^┴^
Prevention of atopic dermatitis	NC/Nga mice	1.5 mg/kg	IgE ^↓^

^↑^ Upregulation; ^↓^ downregulation; ^┴^ inhibition.

**Table 3:** Composition variation of essential oil from various parts of *C. sativum*.

Plant Parts	Compounds	Percentage Composition
Seeds	Linalool	58.0–80.3
	γ-terpinene	0.3–11.2
	α-pinene	0.2–10.9
	p-cymene	0.1–8.1
	Camphor	3.0–5.1
	Geranyl acetate	0.2–5.4
Flower	Benzofuran,2,3-dihydro	15.4
	Hexadecanoic acid, methyl ester	10.32
	2,4a-epioxy-3,4,5,6,7,8,-hexahydro-2,5,5,8a-tetramethyl-2h-1-benzofuran	9.35
	2-methoxy-4-vinylphenol	8.8
	2,3,5,6-tetrafluroanisole	8.62
	2,6-dimethyl-3- aminobenzoquinone	6.81
	Dodecanoic acid	5%
Leaves	Decanal	19.09
	*Trans*-2-decenal	17.54
	2-decen-1-ol	12.33
	Cyclodecane	12.15
	*Cis*-2-dodecena	10.72
	Dodecanal	4.1
	Dodecan-1-ol	3.13

Information in the table was adapted from Mandal et al. [66].

## Data Availability

Not applicable.
